# Increased Brain Iron Deposition in the Putamen in Patients with Type 2 Diabetes Mellitus Detected by Quantitative Susceptibility Mapping

**DOI:** 10.1155/2020/7242530

**Published:** 2020-09-25

**Authors:** Jing Li, Qihao Zhang, Nan Zhang, Lingfei Guo

**Affiliations:** ^1^Department of Radiology, Beijing Friendship Hospital, Capital Medical University, Beijing, China. 95 Yongan Road, Xi Cheng District, Beijing 100050, China; ^2^Department of Radiology, Weill Cornell Medical College, New York. 71st E No. 515, 10044 New York, USA; ^3^Shandong Medical Imaging Research Institute, Cheeloo College of Medicine, Shandong University, Jinan, Shandong, China. Jing-wu Road No. 324, Jinan, Shandong 250021, China

## Abstract

**Background:**

The underlying brain structural changes in type 2 diabetes mellitus (T2DM) patients have attracted increasing attention. The insulin-resistant state causes iron overload in neurons and leads to lesions in the central nervous system. Quantitative susceptibility mapping (QSM) can provide a noninvasive quantitative analysis of brain iron deposition. We aimed to compare the difference of brain iron deposition in the gray matter nucleus between T2DM patients and healthy elderly individuals using QSM.

**Methods:**

Thirty-two T2DM patients and thirty-two age- and gender-matched healthy controls (HCs) were enrolled in this research. Twenty-three patients and twenty-six HCs underwent cognitive assessments. Brain QSM maps were computed from multiecho GRE data using morphology-enabled dipole inversion with automatic uniform cerebrospinal fluid zero reference algorithm (MEDI+0). ITK-SNAP was used to measure the susceptibility values reflecting the content of iron in the regions of interest (ROIs).

**Results:**

The study included thirty-two T2DM patients (20 males and 12 females; mean age of 61.09 ± 9.99 years) and 32 HCs (14 males and 18 females; mean age of 59.09 ± 9.77 years). These participants had no significant difference in age or gender (*P* > 0.05). Twenty-three patients with T2DM (11 males and 12 females; mean age, 64.65 ± 8.44 years) and twenty-six HCs (14 males and 12 females; mean age, 62.30 ± 6.13 years) received an assessment of cognitive function. T2DM patients exhibited an obviously (*t* = 3.237, *P* = 0.003) lower Montreal Cognitive Assessment (MoCA) score (26.78 ± 2.35; HCs, 28.42 ± 0.64; normal standard ≥26) and a higher Stroop color-word test (SCWT)-C score [87(65,110); HC, 63(60,76.75), *Z* = −2.232, *P* = 0.003] than HCs. The mean susceptibility values in the putamen appeared obviously higher in T2DM patients than in HCs (*t* = −3.994, *P* < 0.001). The susceptibility values and cognitive assessment scores showed no obvious association (*P* > 0.05). However, an obvious correlation was observed between the changes in the susceptibility values in the putamen and the thalamus/dentate nucleus (*r* = 0.404, *P* < 0.001; *r* = 0.423, *P* < 0.001).

**Conclusion:**

T2DM patients showed increased susceptibility values in the putamen and had declines in executive functions, but the linear association between them was not statistically significant. Changes in susceptibility values in the putamen indicated increased iron deposition and might be used as a quantitative imaging marker of central nervous system injury in T2DM patients. QSM might be able to help probe micro neuronal damage in gray matter and provide information on diabetic encephalopathy.

## 1. Background

As a complicated metabolic disorder, diabetes mellitus (DM) mainly features hyperglycemia caused by insulin insufficiency and dysfunction. According to figures, approximately 425 million adults suffered DM in 2017 worldwide [1, 2]. Type 2 diabetes mellitus (T2DM) is a long-term DM, and its symptoms include hyperglycemia, insulin resistance, and insulin deficiency, which accounts for the great majority of the diabetes burden, comprising 85% of DM patients. In T2DM, peripheral insulin resistance and insulin compensatory hypersecretion from the pancreatic islets are likely to decrease before the decrease in islet secretory function leads to several complications, such as neuropathy, nephropathy, atherosclerosis, and retinopathy [3].

Insulin resistance in the brain causes follow-up sequelae that possibly contribute to tau hyperphosphorylation and/or amyloid accretion. Insulin comes into play by distributing iron to neuronal tissue; however, the insulin-resistant state disrupts this process, and as a result, iron is overloaded in neurons and finally becomes detrimental [4]. T2DM can impact diseases of the peripheral nervous system, and excessive iron could lead to lesions in the central nervous system. T2DM patients suffer cognitive deficits in memory, executive function, attention, visuospatial abilities, and deficits in other domains. It has been found that T2DM patients show poor performance on the cognitive scale, and their manifestation of neural slows and cortical atrophy increases [5]. It has been proposed that the early onset of T2DM, weak glycemic control, and the existence of microvascular and macrovascular complications can together result in cognitive impairment. T2DM can be used independently to measure the risk of Alzheimer's disease (AD), vascular dementia (VD), and mild cognitive impairment (MCI) [6]. Patients with T2DM show more executive dysfunction than nondiabetics [7], which is associated with mild-to-moderate executive function (EF) decrements [8]. EF declines in T2DM patients compared to healthy older controls but to an extent smaller than that suffered by AD patients; the impairment of executive processing of T2DM patients suggests that these patients are at risk of developing AD [9].

Iron is an important auxiliary factor for the body's oxygen binding and transportation, energy, and material metabolism, which can affect oxygen transportation, cell growth regulation, electron transport, and synthesis of DNA. If iron homeostasis is impaired, reactive oxygen species will be produced excessively, and apoptosis will occur [10, 11]. In addition, as iron accumulates, protein will undergo misfolding and aggregation, resulting in different related diseases. Iron chelation therapy can reduce the overload of iron, which accordingly has the function of changing the glycemic control of T2DM individuals [12].

Based on an increasing number of studies regarding magnetic resonance imaging (MRI), brain iron overload can be seen in different neurodegenerative diseases [13, 14]. Quantitative susceptibility mapping (QSM) is a newly found MRI approach that can help quantify materials with changing susceptibility and has been shown to provide a noninvasive quantitative analysis of brain iron deposition [15–17]. The brain is the most metabolically active organ in the body and has a high demand for iron [18]. A previous study reported that the pallidum, putamen, substantia nigra, red nucleus, and caudate nucleus most likely have high concentrations of nonheme iron [19]. Iron is known to be the main source of susceptibility values in the deep nuclei regions [20]. Susceptibility values calculated from QSM have been reported to have a strong correlation with iron concentration in the human brain, especially in deep gray matter structures [21]. The reasons for the differential regional distribution of brain iron may be related to the iron ions being involved in the synthesis of neurotransmitters and the various metabolic activities across regions and types of neurons. However, the association between cerebral iron accumulation and T2DM in vivo in patients with T2DM has not been completely elucidated. The association between iron accumulation and cognitive decline in T2DM is not known. In this study, the noninvasive quantitative analysis QSM was performed to assess the deposition of brain iron in T2DM patients. To explore the differences between the patients with T2DM and healthy elderly individuals, QSM was used to evaluate and compare the deposition characteristics of iron in the gray matter nuclei in T2DM patients compared to healthy volunteers.

## 2. Methods

### 2.1. Participants

In the cross-sectional study, thirty-two T2DM patients (20 male; mean age, 61.09 ± 9.99 years; range, 39-75 years) and thirty-two age- and gender-matched healthy control volunteers (HCs) (14 male; mean age, 59.09 ± 9.77 years; range, 35-73 years) were enrolled from September 2018 to August 2019. All patients met the diagnostic criteria of T2DM (diagnosed according to American Diabetes Association criteria). For this study, there was no special selection of people with T2DM based on metabolic control, the presence of micro- or macrovascular complications, neuropathy, duration of disease or type of treatment for hyperglycemia, presence of vascular risk factors, or arterial hypertension. The healthy control volunteers without T2DM did not have a history of elevated blood glucose levels, and their blood glucose levels at inclusion were within the normal range (fasting glucose <5.5 mmol/l). For inclusion, participants had to be between 55 and 80 years, have a history of T2DM for longer than 1 year, and be willing to undergo an MRI scan. Exclusion criteria were a history of psychiatric or neurological disorders (including cerebrovascular accidents) that might influence cognitive functioning, a history of alcohol or substance abuse, acute complications of T2DM (ketoacidosis, severe hypoglycemia) within 3 months preceding the examination, and an MRI scan contraindication. This study obtained approval from the institutional review board of Shandong Medical Imaging Research Institute Affiliated to Shandong University. All participants were informed of the detailed experimental procedures and signed informed consent forms. Considering that the participants may have cognitive impairment, all subjects were invited to test their levels of cognitive function, and 49 people (23 T2DM patients and 26 HC) completed the questionnaire.

### 2.2. Clinical Data Collection

Participants underwent a detailed interview and clinical examination. Age, level of education (number of years in primary school, high school and university), diabetes duration, and medical treatment were recorded. Diabetic retinopathy was diagnosed by an ophthalmological examination. Chronic kidney disease was diagnosed based on the presence of micro- or macroalbuminuria and/or a decreased glomerular filtration rate (GFR) <60 ml/min. The assessment of albuminuria was performed by measurement of the urinary albumin-to-creatinine ratio (UACR) in a random spot urine collection. Microalbuminuria was defined as a UACR of 30–299 mg/g creatinine, and macroalbuminuria was defined as a UACR of >300 mg/g creatinine in two repeat measurements. Measurements of systolic and diastolic BP were performed. Arterial hypertension was defined as an average systolic BP >140 mmHg, a diastolic BP >90 mmHg, or self-reported use of blood pressure-lowering medication. Fasting glucose, HbA1c fasting triglycerides, and fasting cholesterol were determined through laboratory examination of venous blood samples. Weight and height were measured, and BMI was calculated. Participants had to attend the clinic on separate days for the cognitive tests and the MRI scan. Assessment of cognitive functioning, collection of venous blood samples, and clinical examination were performed on the same day, whereas the MRI scan was performed on another day. The interval between neurophysiological testing and the MRI scan was 1-4 days. The severity of cerebral small vessel disease (CSVD) in patients was assessed by simple small vessel disease (SVD) scores from clinical MRI scans [22].

### 2.3. Neuropsychological Tests

Standardized general and detailed neurological examinations were performed on the participant. Forty-nine people (23 T2DM patients and 26 HC) underwent the cognitive assessment, and the assessment tools included the Montreal Cognitive Assessment (MoCA) and the Stroop color-word test (SCWT). The MoCA is a one-page total 30-point test administered in 10 minutes [23, 24]. The optimal cutoff points were 13/14 for illiterate individuals, 19/20 for individuals with 1 to 6 years of education, and 24/25 for individuals with 7 or more years of education, whose scores below these cut-off values indicate cognitive impairment [25]. The SCWT was used to evaluate basic human executive function, particularly attention and informational processes [26]. This task consists of the presentation of colors printed on either neutral words or incongruent color words. In this test, participants were first asked to say the name of the color given in black wording (card A) and then the color of the stimulus (card B). Card C featured the names of colors but in a competing color name (e.g., the word “blue” written in red). Scores were derived from times taken by participants to complete each part (A, B, C), the sum of multiple subtests (A, B, C), and the difference in completion times between card C and card B.

### 2.4. Image Acquisition

All subjects were imaged on a MAGNETOM Skyra 3.0 T MR scanner (Siemens Healthcare, Erlangen, Germany) using a 32-channel head coil for signal reception. The brain scanning protocol consisted of a 3D T1-weighted (T1W) magnetization prepared rapid gradient echo (MPRAGE) sequence for anatomic structure (TR = 7.3 ms, TE = 2.4 ms, TI = 900 ms, flip angle = 9°, isotropic voxel size = 1 mm3) and a 3D multiecho gradient echo (ME-GRE) sequence for QSM (TR = 50 ms, first TE = 6.8 ms, TE interval = 4.1 ms, number of echoes = 10, flip angle = 15°, voxel size = 1 × 1 × 2 mm3). In addition, T2-weighted (T2W) turbo spin echo (TSE), T2W fluid-attenuated inversion recovery (FLAIR), diffusion-weighted, and SWI were acquired to detect brain abnormalities.

### 2.5. QSM Preprocessing and Quantitative Analysis

Brain QSM maps were computed from ME-GRE complex image data using morphology-enabled dipole inversion with an automatic uniform cerebrospinal fluid (CSF) zero reference algorithm (MEDI+0) [27]. Briefly, a nonlinear fitting of the multiecho data was performed to estimate the total field, followed by spatial field unwrapping and background field removal using the projection onto dipole fields (PDF) algorithm to compute the local field, which was then inverted to obtain the final susceptibility map. Structural priors (edges) derived from the magnitude image and a regularization term enforcing uniform susceptibility distribution of the CSF within the lateral ventricles were used in the numerical inversion to improve QSM quality and to provide CSF as an automatic susceptibility reference. The CSF mask was determined by thresholding the R2∗ map computed from the GRE magnitude data and imposing voxel connectivity [27].

**Figure 1:**
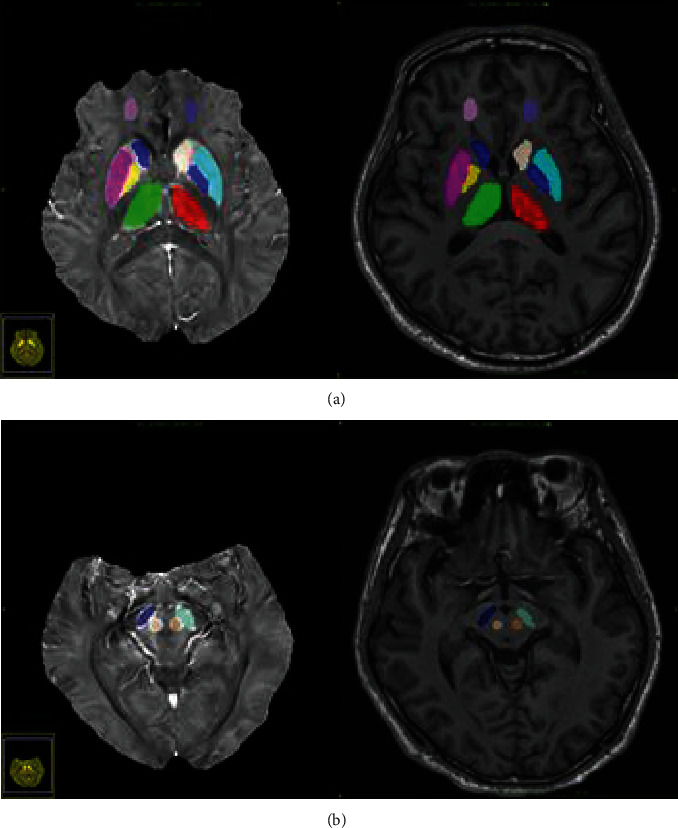
ROI sketch diagram. The 3D T1WI and QSM images were coregistered to a magnitude image of the first echo acquired from the 3D GRE sequence of the same subject by using FSL software. The gray matter nuclei and the frontal white matter (ROIs larger than 150 voxels) were drawn entirely by hand. The average QSM value in each ROI was then computed from all voxels overlapping with the corresponding label.

The conventional images (T1W, T2W, FLAIR) were processed with an FMRIB Software Library (FSL) automated pipeline, which consisted of brain extraction using BET, bias field correction using FAST, and linear coregistration to the GRE magnitude image (which is in the same space as QSM) using FLIRT with six degrees of freedom. For region of interest (ROI) analysis, the subcortical structures that are closely related to cognitive, emotional, and motor functions (thalamus, caudate nucleus, putamen, pallidum, red nucleus, substantia nigra, dentate nucleus) were traced directly on the QSM images by a neuroradiologist with 16 years of experience using ITK-SNAP v3.8 software. The details are shown in Figure 1. The mean susceptibility value of the gray matter nucleus minus the mean susceptibility value of the white matter in the frontal lobe of the same patient was calculated. Separate left and right ROIs were obtained for each brain structure. The mean susceptibility value within each ROI was then recorded.

### 2.6. Statistical Analysis

The Statistical Package for the Social Sciences (Version 21.0 for Windows; SPSS, Chicago, Ill) helped to carry out statistical analysis. First, a descriptive analysis of thirty-two T2DM patients and thirty-two HCs was performed. The measurement data were represented in the form of the mean ± standard deviation or median and interquartile range if the data were not normally distributed. The counting data were represented in the form of *n* (%). The chi-square test was applied to the comparison of count data. To compare the susceptibility values within a specific ROI or cognitive assessment scores between the patients with T2DM and HCs, the independent sample *t*-test or the Mann–Whitney *U* test were used. Because multiple hypotheses were tested, we used the Bonferroni method for correction to avoid type-I error. The correlations between susceptibility values and cognitive function scores were determined via Pearson or Spearman correlation analysis.

## 3. Results

### 3.1. Participant Characteristics

**Figure 2:**
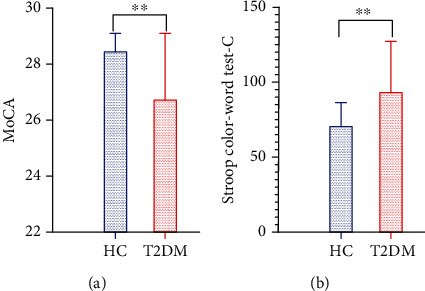
Differences between MoCA and SCWT scores in patients with T2DM and HCs.

Thirty-two patients with T2DM (20 males and 12 females, with a mean age of 61.09 ± 9.99 years) and 32 HCs (14 males and 18 females, with a mean age of 59.09 ± 9.77 years) were included in this study. These participants showed no significant difference in age, gender, and education level between groups (*t* = 1.650, *P* = 0.205; *t* = 0.177, *P* = 0.674; *t* = 0.515, *P* = 0.609). Of the sixty-four participants, twenty-three patients with T2DM (11 males and 12 females; mean age, 64.65 ± 8.44 years) and twenty-six HCs (14 males and 12 females; mean age, 62.30 ± 6.13 years) received an assessment of cognitive function.  Table 1 lists the clinical characteristics of the participants in the study. T2DM patients exhibited an obviously (*t* = 3.237, *P* = 0.003) lower MoCA score (26.78 ± 2.35; HCs, 28.42 ± 0.64; normal standard ≥26) and higher SCWT-C score [87(65,110)] than HCs [63(60,76.75)] (*Z* = −2.232, *P* = 0.003). The scores for each cognitive and behavioral assessment subindex in these tests and evaluations are shown in Table 2 and Figures 2(a) and 2(b).

### 3.2. Susceptibility Value Analysis across ROIs

**Figure 3:**
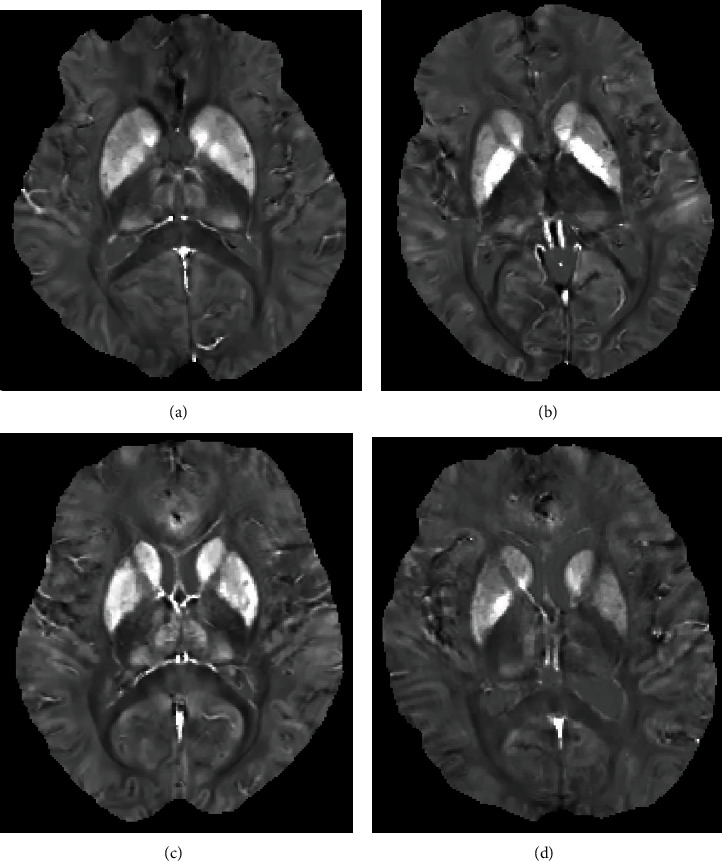
Brain susceptibility value differences between the patients with T2DM and HCs. (a) Patients with T2DM, male, 67 years, DM history for 15 years, (b) HC, female, 66 years. (c) Patients with T2DM, female, 65 years, DM history for 18 years, (d) HC, female, 64 years.

**Figure 4:**
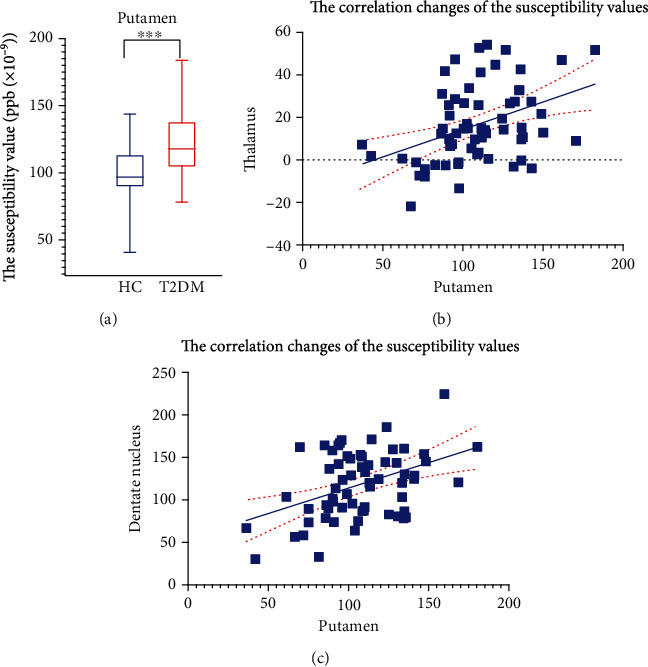
(a) The susceptibility value [ppb (×10^−9^)] differences between the patients with T2DM and HCs in the gray matter nuclei. (b, c) The correlation between changes in susceptibility values between putamen and thalamus/dentate nucleus.

The comparison of susceptibility values from the patients with T2DM and the HCs is shown in Table 3 and Figure 3. To avoid type-I error, Bonferroni correction was adopted, and the new *P* value level was 0.007. We found that T2DM patients presented obviously higher mean susceptibility values than HCs in the putamen (*t* = −3.994, *P* < 0.001; Figure 4(a)). However, the differences between the patients with T2DM and HCs were not significant in the thalamus (*t* = −1.803, *P* = 0.076), pallidum (*t* = −0.242, *P* = 0.809), caudate nucleus (*t* = −0.756, *P* = 0.452), red nucleus (*t* = −1.153, *P* = 0.253), substantia nigra (*t* = −0.245, *P* = 0.808), and dentate nucleus (*t* = −2.301, *P* = 0.025). The susceptibility values in the patients with T2DM tended to be higher in most gray matter nuclei than those in the HCs. In the patients with T2DM and HCs, the susceptibility values on the left side of the ROIs were not significantly higher than those on the right side (*P* > 0.007), as shown in Table 4. The susceptibility values and cognitive assessment scores showed no obvious association (*P* > 0.05). However, an obvious correlation was observed between the changes in the susceptibility values in the putamen and the thalamus/dentate nucleus (*r* = 0.404, *P* < 0.001; *r* = 0.423, *P* < 0.001; Figures 4(b) and 4(c)).

## 4. Discussion

Iron is a fundamental requirement for most known life forms and is the richest trace element in the human body [28]. Iron acts as a significant component of hemoglobin that participates in oxygen transport. Iron in the nervous system can also affect catecholamine neurotransmitter metabolism and myelin formation. Excessive deposition of iron in the brain of the aged will easily lead to different kinds of neurodegenerative diseases [29]. Therefore, studying and understanding the iron metabolism mechanism in the brain together with its regulation are of vital significance [30]. An example of misregulation of iron metabolism resulting in functional iron deficiency was shown in animals with targeted deletions of iron regulatory protein 2 (IRP2). Abnormal accumulations of ferric iron were detected in the cell bodies of oligodendrocytes and in their extensions. Axonal degeneration was present in areas in which increased ferric iron was detected by iron stains, and it initially seemed likely that the iron detected within the axonal framework, which was mainly sequestered in ferritin, could be a cause of axonal degeneration, resulting in loss of neurofilament structures and collapse of the axon [31]. Because deep gray matter nuclei contain important structures closely involved in cognitive, emotional, and motor functions, this study selected these areas as research targets. The relationship between iron metabolism and T2DM appears to be bidirectional. Iron can affect glucose metabolism via its deleterious effect on pancreatic cells, and glucose metabolism can impair several iron metabolic pathways [32]. The overload of brain iron could result in insulin resistance together with cognitive decrease in animal obesity models and human obesity models [33]. Therefore, we speculated that brain iron deposition would increase in T2DM patients, and our study results confirmed this. In this study, we found that the brain iron deposits in patients with T2DM have an increasing trend compared with healthy elderly individuals in iron-rich gray matter nuclei, and the regional susceptibility values in the putamen of patients had significant differences compared with HCs. A previous study also reported that T2DM patients exhibited significantly increased susceptibility in some brain regions [34]. The precise mechanisms underlying the higher iron concentration in T2DM are not understood. According to a review of the current literature, there are several potential reasons for cerebral iron deposition in patients with T2DM. Iron binds to amyloid-*β* to catalyze prooxidant radicals to be produced, thereby increasing the toxicity of the peptide, which binds to tangles as well, leading to the formation of toxic radicals in neurons in a similar way [35]. Synthetic amyloid-*β* intoxication in the mouse brain causes tau-dependent iron accumulation as well as cognitive impairment [36], which demonstrates that tau can mediate the effects of iron.

Our results illustrated that the putamen is the most significant change region in T2DM patients. Previous studies have shown that isolated putamen hemorrhage can lead to impaired frontal lobe function in patients, leading to attention-executive dysfunction [37]; there are also task-related attentional and executive function disruptions involving the putamen of patients with multiple sclerosis clinically isolated syndrome [38, 39]. Changes in the putamen are also very important to T2DM patients. For example, diabetic striatopathy (DS) is characterized by basal ganglia changes, which are visualized as hyperintense on T1-weighted MR images [40], and the most common pattern of striatal anomalies of DS are isolated putamen involvement, followed by combined caudate nucleus-putamen involvement. The cause of hyperintensity on T1-weighted MR images is still unknown, but some pathological analyses could directly shed light on the pathogenesis of DS-associated striatal lesions. According to the six available pathology reports, the reactive astrocytosis documented in five pathology reports and abundant gemistocytes in another biopsy may partially explain the striatal hyperintensity on T1-weighted MRI but not hyperdensity on CT. However, only two pathology reports showed calcium deposits or punctuate calcification. In contrast, microvascular hemorrhage may be probable based on the findings of four pathological analyses showing hemosiderin-containing macrophages, microhemorrhage, extravascular hemosiderin deposits, and erythrocyte extravasation [40]. “Physiologic calcification” in the basal ganglia (including the putamen) is always accompanied by the deposition of other metals (e.g., zinc, iron, copper, manganese, and aluminum) [41]. We found that T2DM patients presented obviously higher mean susceptibility values in the putamen than HCs, which may be explained by the phenomenon that although calcium in these areas causes negative susceptibility on QSM, this may be overwhelmed by the strong paramagnetic deposits, such as iron, because QSM measures overall bulk magnetic susceptibility [42]. Meanwhile, microbleeds change local magnetic susceptibility because of the pathologic iron accumulation [43]. QSM demonstrated excellent accuracy and reliability in detecting microbleeds [44]. Our findings also supported that microvascular hemorrhage may be responsible for the hyperintensity on T1-weighted images.

The basal ganglia play important roles in various cognitive phenomena, including executive functions, working memory, implicit and explicit learning and memory, adaptive motor control, and sensorimotor learning [45]. The cognitive and motor functions of the basal ganglia are qualitatively accounted for by classic models of parallel functionally segregated basal ganglia-thalamocortical circuits, elements of which include discrete parts of the striatum (putamen, caudate nucleus, and pallidum), substantia nigra, thalamus, and cortex. The striatum has various brain functions, including motor control and learning, language, reward, cognitive functioning, and addiction through functional cortico-striato-thalamocortical neural pathways [46, 47]. Therefore, a pathologic state in the striatum can lead to a broad range of clinical manifestations from motor dysfunction, such as Parkinson's disease, to various psychiatric disorders [48]. Different parts of the striatum receive afferent inputs from different cortical regions and project their efferent output to the cortex through the thalamus [49, 50]. The thalamus is a relay and integration center connecting subcortical and cortical regions, thereby playing crucial roles in awareness, sensory, motor, and cognitive functions. The dentate nucleus is the most lateral deep cerebellar nucleus and is rich in iron [51]. The dentate nucleus is capable of regulating fine control regarding voluntary movements, language, cognition, and sensory functions. Utilizing the dentatothalamic tract, the dentate nucleus sends output signals through the ipsilateral superior cerebral peduncle and then decussates to synapse in the contralateral ventrolateral (VL) thalamic nucleus. VL neurons send fibers to the precentral gyrus, premotor cortex, prefrontal gyri, posterior parietal areas, and basal ganglia, specifically the putamen [52, 53]. The basal ganglia and cerebral cortex form an integrated network that is topographically organized such that the motor, cognitive, and affective territories of each node in the network are interconnected [53, 54]. In a study of the relationship between QSM and cognitive impairment in type 2 diabetes patients, correlations of the basal ganglia, thalamus, red nucleus, and substantia nigra with neuropsychological cognitive scores were found [39]. We also found significant correlations between the putamen and the thalamus/dentate nucleus in iron deposition changes in the participants. It was hinted that the synergy of these changes may potentially affect the cortico-striato-thalamocortical neural pathways and impact the neural function of the brain, which may affect voluntary movements, cognition, language, and sensory functions.

Few studies have examined the relationship between the deposition of brain iron and cognitive function in T2DM patients. We intended to identify the relationship between iron accumulation and central nervous system injury in patients with T2DM. A previous study investigated the deposition of iron in the brain in T2DM patients with MCI using QSM and related the susceptibility value with cognitive impairments. They found that susceptibility values in the left putamen increased and were significantly related to the neuropsychological cognitive score [39]. Our study showed similar results, which also suggested that there were obviously higher susceptibility values in the putamen in T2DM patients than in healthy elderly individuals. The comparison of the MoCA and SCWT scores between the patients with T2DM and healthy elderly individuals demonstrated that the patients with T2DM showed mild cognitive impairment (MCI) compared to the healthy elderly individuals. MCI is generally considered to be the precursor of AD. Our results suggested that the dentate nucleus-thalamus-putamen is the pivotal part of the cortico-striato-thalamocortical neural pathways that might predict the conversion of MCI to AD in patients with T2DM. The increase in susceptibility values can be used as an important quantitative imaging marker. Previous studies have also shown that isolated putamen hemorrhage can lead to impaired frontal lobe function in patients, leading to attention-executive dysfunction [37]; there are also task-related attentional and executive function disruptions involving the putamen of patients with multiple sclerosis clinically isolated syndrome [38, 39]. However, we did not find correlations between susceptibility values and cognitive function scores. This difference may be explained by the selection of different research samples or cognitive assessment scores influenced by many factors.

QSM acts as a new MRI approach that can quantify materials with changing susceptibility and has been shown to provide a noninvasive quantitative analysis of brain iron deposition [15, 16]. A study reported that the reproducibility of susceptibility values in human subjects over 2 time points was excellent for both 3 T and at 1.5 T MR scanners and for different algorithm methods [21]. This excellent reproducibility and consistency indicate that the magnetic susceptibility of a given object is theoretically an invariable value and that QSM is a reliable quantification method to measure magnetic susceptibility longitudinally between different time points and between different magnets. QSM exhibits a stronger selectivity for iron than T2∗ relaxometry and can analyze data obtained via standard sequence acquisition, which are available for a majority of commercial scanners. QSM acts as a useful computer algorithm for deriving sensitivity values to iron levels from appropriate MRI data [17, 55]. However, QSM detects paramagnetic substances, such as Zn2+, Cu2+, Ca2+, Fe3+, and gadolinium ions, and several studies have reported that the magnetic susceptibility of the globus pallidus and dentate nucleus increased after serial administration of gadobutrol [56, 57]. The high abundance and high susceptibility of cellular iron compounds make iron the major biometal source for tissue QSM [17]. Brain iron overload (more than the amount that can be safely transported and stored) is a cause and/or cofactor of a variety of neurodegenerative diseases, including AD and Parkinson's disease [58, 59]; therefore, QSM technology can be used to measure biometal changes during pathogenesis, progression, and treatment in many neurodegenerative diseases.

The study was a preliminary cross-sectional design study of brain iron changes in T2DM patients in a relatively small sample size. The iron deposition dynamics shall be observed, together with the examination of longitudinal levels of brain iron in T2DM patients in larger samples at different stages. It is necessary to perform a prospective study covering a large scale for determining the changes of magnetic susceptibility in certain regions as well as further exploring the potential mechanisms and the effect posed by iron deposition in gray matter nuclei pathology. Although automatic segmentation is the most appropriate method specific for imaging analysis based on previous studies, in this study, we use manual segmentation as the reference standard for complicated structures and try to use methods of whole-brain voxel analysis to make comparisons in further research.

## 5. Conclusion

In conclusion, T2DM patients showed increased iron deposition in the putamen. Cerebral iron deposition exacerbates the decline in cognitive function (executive function) in patients with T2DM, but the linear association was not statistically significant. This study found that the deposition of iron in the brain exhibits an association with T2DM, and the association may greatly affect the T2DM process. Changes in susceptibility values in the putamen indicated increased iron deposition and might be used as a quantitative imaging marker of central nervous system injury in T2DM patients. QSM might be able to help probe micro neuronal damage in gray matter and provide information on diabetic encephalopathy.

## Figures and Tables

**Table 1:** Clinical characteristics of the participants.

	T2DM (*n* = 32)	HC (*n* = 32)	Statistical value (*x*^2^ or *t*)	*P*
Gender (male)	14 (43.75%)	20 (62.50%)	2.259	0.133
Age (years)	61.09 ± 9.99	59.09 ± 9.77	-0.809	0.421
Risk factors for cardiovascular disease				
BMI (kg/m2)	27.5 ± 5.6	25.3 ± 4.7	1.315	0.104
Fasting serum cholesterol (mmol/l)	5.4 ± 1.1	5.3 ± 0.9	0.398	0.634
Fasting serum triglycerides (mmol/l)	2.6 ± 0.8	2.2 ± 0.6	1. 324	0.117
Hypertension	15 (46.88%)	13 (40.62%)	0.254	0.614
Use of antihypertensive medication	12 (37.50%)	11 (37.50%)	0.068	0.794
History of myocardial infarction	0	0		
SVD scores	1.3 ± 0.45	1.1 ± 0.34	1.291	0.208
Type 2 diabetes-related factors				
Fasting plasma glucose (mmol/l)	9.2 ± 2.44	5.2 ± 1.26	3.551	<0.001
HbA1c (mmol/mol)	61.1 ± 10.3	—		
HbA1c (%)	7.9 ± 1.3	—		
Diabetes duration (years)	11.2 ± 6.5	—		
Use of insulin	16 (50.00%)	—		
Type 2 diabetes microvascular complications				
Retinopathy	7 (21.87%)	—		
Chronic kidney disease	2 (6.25%)	—		
Peripheral somatic neuropathy	8 (25.00%)	—		
Autonomic cardiovascular neuropathy	5 (15.62%)	—		

a: Chi-square test; b: independent samples *t*-test; c: Mann–Whitney *U* test.

**Table 2:** Cognitive functioning assessment of the participants.

Variables	T2DM (*n* = 23)	HC (*n* = 26)	Statistical value	*P*
Gender (male)	11 (47.82%)	14 (53.84%)	0.177^a^	0.674
Age, years	64.65 ± 8.44	62.30 ± 6.13	1.650^b^	0.205
Education, years	11.34 ± 2.26	11.69 ± 2.40	0.515 ^b^	0.609
MoCA	26.78 ± 2.35	28.42 ± 0.64	3.237 ^b^	0.003
SCWT-A	24 (21,30)	24 (20,26.25)	-2.205	0.360
SCWT-B	40 (35,50)	34 (30,37.75)	-0.915	0.026
SCWT-C	87 (65,110)	63 (60,76.75)	-2.232	0.003
SCWT (C-B)	48 (29,58)	34 (25.75,40.5)	-2.963	0.027
SCWT-Sum (A+B+C) #	157 (128,188)	123 (112,152)	-2.336c	0.020

a: Chi-square test; b: independent samples *t*-test; c: Mann–Whitney *U* test. ^∗^scores measured as a number; ^#^scores measured in seconds. Sum: the sum of multiple subtests.

**Table 3:** The susceptibility value differences [ppb (×10^−9^)] in gray matter nuclei.

Variables	T2DM (*n* = 32)	HC (*n* = 32)	Statistical value	*P*
Thalamus	20.17 ± 19.30	12.25 ± 15.64	-1.803	0.076
Pallidum	198.78 ± 42.08	196.18 ± 43.51	-0.242	0.809
Putamen	121.60 ± 25.37	96.43 ± 25.03	-3.994	<0.001
Caudate nucleus	94.89 ± 44.18	88.29 ± 22.01	-0.756	0.452
Red nucleus	179.18 ± 36.59	168.55 ± 37.11	-1.153	0.253
Substantia nigra	179.93 ± 42.89	177.38 ± 40.58	-0.245	0.808
Dentate nucleus	129.96 ± 33.86	108.02 ± 41.98	-2.301	0.025

^∗^Bonferroni correction, the new significance level was *P* < 0.05/7 = 0.007.

**Table 4:** Susceptibility value differences [ppb (×10^−9^)] between the left and right in gray matter nuclei.

Variables	Mean and standard deviation	Standard error	95% confidence interval		t	*P*
			Lower limit	Upper limit		
Thalamus (left-right)	−0.96 ± 17.84	2.23	-5.41	3.49	-0.431	0.668
Pallidum (left-right)	−2.10 ± 21.53	2.69	-7.48	3.27	-0.782	0.437
Putamen (left-right)	−0.09 ± 21.76	2.72	-5.52	5.34	-0.034	0.973
Caudate nucleus (left-right)	−4.81 ± 55.21	6.90	-18.60	8.98	-0.697	0.488
Red nucleus (left-right)	−3.38 ± 28.91	3.61	-10.60	3.83	-0.937	0.352
Substantia nigra (left-right)	2.72 ± 26.97	3.37	-4.00	9.46	0.809	0.421
Dentate nucleus (left-right)	5.23 ± 19.28	2.41	0.41	10.05	2.170	0.034

Left-right: the susceptibility value in the left region after subtracting the susceptibility value in the right region.^∗^Bonferroni correction, the new significance level was *P* < 0.05/7 = 0.007.

## Data Availability

This statement should describe how readers can access the data supporting the conclusions of the study and clearly outline the reasons why unavailable data cannot be released.
